# Impact of Frailty Upon Surgical Decision-Making for Left-Sided Colon Cancer

**DOI:** 10.31486/toj.22.0120

**Published:** 2023

**Authors:** Udai S. Sibia, Shivani B. Badve, Alexandra C. Istl, J. Robert Klune, Adam I. Riker

**Affiliations:** ^1^ Department of Surgery, Anne Arundel Medical Center at Luminis Health, Annapolis, MD; ^2^ Division of Surgical Oncology, Medical College of Wisconsin, Milwaukee, WI

**Keywords:** *Anastomosis–surgical*, *colorectal surgery*, *colostomy*, *frailty*, *postoperative complications*

## Abstract

**Background:** Frailty is characterized by reduced physiologic reserve, and for patients with colon cancer, frailty is associated with increased morbidity after resection. One commonly cited reason for performing an end colostomy vs a primary anastomosis in left-sided colon cancer is the belief that frail patients do not have the physiologic reserve to withstand the morbidity associated with an anastomotic leak. We explored the impact of frailty on the type of operation performed in patients with left-sided colon cancer.

**Methods:** We queried the American College of Surgeons National Surgical Quality Improvement Program for patients with colon cancer who underwent a left-sided colectomy from 2016 to 2018. Patients were categorized using the modified 5-item frailty index. Multivariate regression was used to identify independent predictors of complications and type of operation performed.

**Results:** Of 17,461 patients, 20.7% were considered frail. Frail patients received an end colostomy more often than nonfrail patients (11.3% vs 9.6%, *P*=0.01). On multivariate analysis, frailty was a significant predictor for total medical complications (odds ratio [OR] 1.45, 95% CI 1.29-1.63) and readmission (OR 1.53, 95% CI 1.32-1.77) but was not independently associated with organ space surgical site infections or reoperation. Frailty was independently associated with receiving an end colostomy vs a primary anastomosis (OR 1.23, 95% CI 1.06-1.44), but an end colostomy did not decrease the risk of reoperation or organ space surgical site infections.

**Conclusion:** Frail patients with left-sided colon cancer are more likely to receive an end colostomy, but an end colostomy does not lower the risk of reoperation or organ space surgical site infections. Based on these results, frailty alone should not prompt the decision to perform an end colostomy, but further studies are needed to guide surgical decision-making in this understudied population.

## INTRODUCTION

Frailty is characterized by decreased physiologic reserve, decreased ability to maintain homeostasis, and decreased tolerance to stressors such as surgery.^1,2^ The Canadian Study of Health and Aging developed a 70-item frailty index to predict mortality and morbidity in patients.^3^ However, this index was difficult to apply in clinical research settings and was therefore adapted using the American College of Surgeons National Surgical Quality Improvement Program (ACS NSQIP) to create the modified frailty index (mFI).^4^ The mFI was originally based on 16 variables mapped to 11 factors but was abbreviated to a validated 5-factor modified frailty index (mFI-5) because variables were removed from the NSQIP user files.^5^ The mFI has been shown to predict morbidity and mortality after surgery across multiple surgical subspecialties.^6-17^ In elective surgery for gastrointestinal cancer, stratifying at 2 or more mFI-11 characteristics has been found to optimally predict increased risk of postoperative morbidity and mortality.^18^

Frailty is common among the elderly and has emerged as an important risk factor for poor outcomes after surgery. In the United States, 53 million people are over the age of 65 years and account for more than 40% of all inpatient surgical procedures.^19,20^ In patients undergoing colorectal surgery for cancer, frailty is an independent predictor of 30-day postoperative outcomes and readmission.^14,15^ Yet little is known about the impact of frailty on surgical decision-making in left-sided colon cancer. This study explored the impact of frailty on the type of operation performed and short-term outcomes in patients undergoing a colectomy for left-sided colon cancer.

## METHODS

Institutional review board approval was obtained for this retrospective analysis of the ACS NSQIP participant user files from 2016 to 2018. Current Procedural Terminology and *International Statistical Classification of Diseases and Related Health Problems, 10th Revision,* codes were used to identify patients undergoing colectomy for left-sided colon cancer. Patients with a diagnosis of rectal cancer and those undergoing a diverting ileostomy procedure were excluded.

The mFI-5 was used to classify patients into frail and nonfrail groups (Table 1). Each factor is assigned 1 point, and the frailty score is the total score divided by 5. Significant frailty is defined as mFI-5 ≥0.4 (2 or more positive factors).

**Table 1.** Factors Included in the 5-Factor Modified Frailty Index

Diabetes mellitus
Congestive heart failure within 30 days prior to surgery
Hypertension requiring medications
History of chronic obstructive pulmonary disease or pneumonia
Partially or totally dependent functional status prior to surgery

The primary aim of this study was to explore the impact of frailty on the type of operation performed (primary anastomosis or end colostomy) in patients with left-sided colon cancer who underwent a left-sided colectomy. The secondary outcome of interest was to identify the impact of frailty on 30-day postoperative outcomes: wound and medical complications, reoperation, and hospital readmission. Wound complications were defined as superficial incisional surgical site infection (SSI), deep incisional SSI, organ space SSI, or wound disruption. Medical complications were pneumonia, unplanned intubation, pulmonary embolism, failure to wean off ventilator >48 hours, progressive renal insufficiency, acute renal failure, urinary tract infection, stroke/cerebrovascular accident, cardiac arrest requiring cardiopulmonary resuscitation, myocardial infarction, transfusion, deep vein thrombosis requiring therapy, and sepsis. Anastomotic leak is not a defined outcome variable in the main ACS NSQIP data user file. Consequently, we used surrogate measures to quantify anastomotic leak: organ space SSI and reoperation.

Descriptive statistics are presented to show the distribution of each outcome of interest. Pearson chi-squared and 2-sided Fisher exact tests were used to analyze differences in categorical variables between the frail and nonfrail groups, and *t* test was used to analyze differences in continuous variables. Univariate analysis was conducted between each outcome of interest and patient characteristic. Outcomes of interest were wound complications, medical complications, readmission, and reoperation. For the multivariate analysis, patient characteristics were age, body mass index (BMI), comorbidities, and frailty. Perioperative risk factors were sepsis within 48 hours prior to surgery, transfusion of ≥1 unit of whole/packed red blood cells within 72 hours prior to surgery, and preoperative serum albumin level <3.5 mg/dL. Emergency surgery and the type of procedure performed (primary anastomosis or end colostomy) were also included in the multivariate analysis. Multivariable logistic regression models were fit to predict each outcome of interest. Variables considered for selection into each model were based on clinical relevance and univariate significance. All analyses were performed using SPSS Statistics, v. 25 (IBM Corporation). An alpha of 0.05 was the threshold for statistical significance.

## RESULTS

A total of 17,461 patients were included in the analysis, of whom 20.7% had an mFI-5 score ≥0.4 (frail) and 79.3% had an mFI-5 score ≤0.2 (nonfrail). Frail patients were older, had a higher BMI, and tended to have more comorbidities than nonfrail patients (Table 2). Smoking and disseminated cancer were more prevalent in the nonfrail group.

**Table 2.** Patient Characteristics, n=17,461

Variable	Frail, n=3,606	Nonfrail, n=13,855	*P* Value
Age, years, mean	69.6	62.9	<0.01
Body mass index, kg/m^2^, mean	31.1	28.4	<0.01
Sex			<0.01
Female	1,486 (41.2)	6,558 (47.4)	
Male	2,120 (58.8)	7,292 (52.7)	
Comorbidities			
Diabetes mellitus	2,888 (80.1)	572 (4.1)	<0.01
Current smoker within 1 year	465 (12.9)	1,924 (13.9)	0.12
Dyspnea	586 (16.3)	689 (5.0)	<0.01
Functionally dependent	408 (11.3)	102 (0.7)	<0.01
Ventilator dependent	8 (0.2)	9 (0.1)	0.01
Chronic obstructive pulmonary disease	639 (17.7)	203 (1.5)	<0.01
Ascites	28 (0.8)	81 (0.6)	0.19
Congestive heart failure within 30 days prior to surgery	225 (6.2)	15 (0.1)	<0.01
Hypertension requiring medications	3,537 (98.1)	5,668 (40.9)	<0.01
Renal failure	19 (0.5)	19 (0.1)	<0.01
Dialysis dependent	60 (1.7)	62 (0.4)	<0.01
Disseminated cancer	348 (9.7)	1,635 (11.8)	<0.01
Steroid use	127 (3.5)	297 (2.1)	<0.01
Weight loss	193 (5.4)	746 (5.4)	0.94
Bleeding disorder	206 (5.7)	412 (3.0)	<0.01
American Society of Anesthesiologists physical status classification score			<0.01
1	2 (0.1)	305 (2.2)	
2	452 (12.5)	5,792 (41.8)	
3	2,587 (71.7)	6,992 (50.5)	
4	552 (15.3)	734 (5.3)	
5	8 (0.2)	12 (0.1)	

Note: Data are presented as n (%) unless otherwise indicated.

Frail patients were more often received as transfers from outside hospital facilities and were more likely to have preoperative hypoalbuminemia, to require preoperative blood transfusion, to require emergency surgery, and to receive an end colostomy instead of a primary anastomosis compared to nonfrail patients (Table 3). Mean hospital stay was longer for frail patients than nonfrail patients (7.7 vs 6.1 days, respectively, *P*<0.01), and frail patients were more likely to be discharged to skilled nursing or rehabilitation facilities than nonfrail patients (16.4% vs 6.7%, respectively, *P*<0.01).

**Table 3.** Perioperative Characteristics

Variable	Frail, n=3,606	Nonfrail, n=13,855	*P* Value
Transfer from an outside hospital facility	179 (5.0)	486 (3.5)	<0.01
Preoperative serum albumin <3.5 mg/dL	924 (25.6)	2,445 (17.6)	<0.01
Sepsis within 48 hours prior to surgery	165 (4.6)	656 (4.7)	0.69
Transfusion^a^	156 (4.3)	299 (2.2)	<0.01
Emergency surgery	926 (25.7)	2,886 (20.8)	<0.01
End colostomy	409 (11.3)	1,330 (9.6)	<0.01
Length of hospital stay, days, mean	7.7	6.1	<0.01
Discharge to skilled nursing or rehabilitation facility	592 (16.4)	924 (6.7)	<0.01

^a^Transfusion of ≥1 unit of whole/packed red blood cells within 72 hours prior to surgery.

Note: Data are presented as n (%) unless otherwise indicated.

Frail patients had higher rates than the nonfrail group of 30-day wound complications (8.4% vs 6.8%, respectively, *P*<0.01), medical complications (22.6% vs 13.6%, respectively, *P*<0.01), reoperation (5.3% vs 4.0%, respectively, *P*<0.01), and readmission (11.0% vs 7.1%, respectively, *P*<0.01) (Table 4). Increasing mFI-5 scores were associated with increasing rates of wound complications, medical complications, reoperation, readmission, discharge to skilled nursing or rehabilitation facility, and median length of stay (Figure). Subset analysis of frail patients revealed no difference in wound complications between patients who received a primary anastomosis vs an end colostomy (Table 5).

**Table 4.** Postoperative Complications

Variable	Frail, n=3,606	Nonfrail, n=13,855	*P* Value
Wound complications			
Superficial incisional SSI	146 (4.0)	411 (3.0)	<0.01
Deep incisional SSI	21 (0.6)	60 (0.4)	0.24
Organ space SSI	132 (3.7)	454 (3.3)	0.25
Wound disruption	35 (1.0)	93 (0.7)	0.06
Any wound complication	302 (8.4)	940 (6.8)	<0.01
Medical complications			
Pneumonia	131 (3.6)	234 (1.7)	<0.01
Unplanned intubation	109 (3.0)	145 (1.0)	<0.01
Pulmonary embolism	26 (0.7)	97 (0.7)	0.89
Ventilator >48 hours	79 (2.2)	148 (1.1)	<0.01
Progressive renal insufficiency	46 (1.3)	76 (0.5)	<0.01
Acute renal failure	28 (0.8)	37 (0.3)	<0.01
Urinary tract infection	76 (2.1)	182 (1.3)	<0.01
Stroke/cerebrovascular accident	17 (0.5)	30 (0.2)	<0.01
Cardiac arrest requiring CPR	44 (1.2)	39 (0.3)	<0.01
Myocardial infarction	56 (1.6)	83 (0.6)	<0.01
Transfusion	440 (12.2)	1,010 (7.3)	<0.01
Deep vein thrombosis requiring therapy	50 (1.4)	125 (0.9)	<0.01
Sepsis	103 (2.9)	368 (2.7)	0.51
Any medical complication	814 (22.6)	1,887 (13.6)	<0.01
Reoperation	192 (5.3)	558 (4.0)	<0.01
Readmission	395 (11.0)	977 (7.1)	<0.01

Note: Data are presented as n (%).

CPR, cardiopulmonary resuscitation; SSI, surgical site infection.

**Figure.**
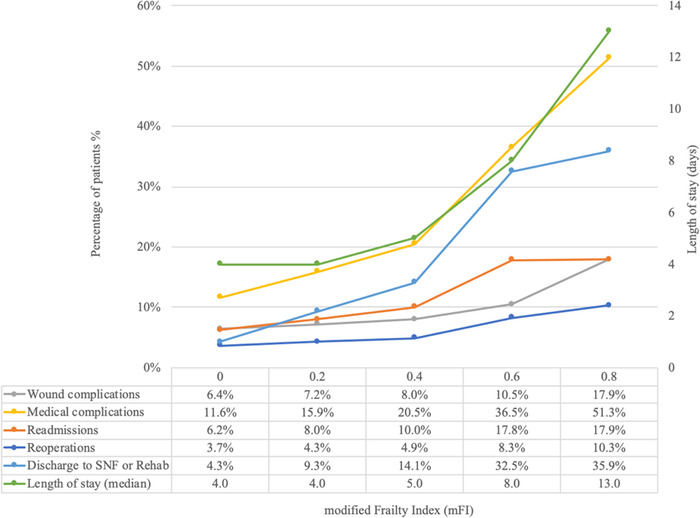
**Association between modified frailty index scores and postoperative outcomes.** Rehab, rehabilitation facility; SNF, skilled nursing facility.

**Table 5.** Postoperative Wound Complications in Frail Patients by Surgery Type

Variable	Primary Anastomosis, n=3,197	End Colostomy, n=409	*P* Value
Superficial incisional SSI	124 (3.9)	22 (5.4)	0.15
Deep incisional SSI	16 (0.5)	5 (1.2)	0.07
Organ space SSI	113 (3.5)	19 (4.6)	0.26
Wound disruption	29 (0.9)	6 (1.5)	0.28

Note: Data are presented as n (%).

SSI, surgical site infection.

Multivariate analysis showed that frailty was independently associated with receiving an end colostomy vs a primary anastomosis (Table 6).

**Table 6.** Multivariate Analysis Identifying Factors Associated With Receiving an End Colostomy

Variable	Odds Ratio	95% CI	*P* Value
Body mass index, ≥30 kg/m^2^	0.80	0.69-0.92	<0.01
Disseminated cancer	2.74	2.36-3.17	<0.01
Preoperative serum albumin <3.5 mg/dL	3.35	2.94-3.81	<0.01
Sepsis within 48 hours prior to surgery	4.65	3.83-5.64	<0.01
Emergency surgery	7.33	6.10-8.81	<0.01
Frailty	1.23	1.06-1.44	<0.01

In the multivariate analysis, frailty was associated with a 45% greater risk for total medical complications (odds ratio [OR] 1.45, 95% CI 1.29-1.63, *P*<0.01) and a 53% greater risk for readmission (OR 1.53, 95% CI 1.32-1.77, *P*<0.01) compared to the nonfrail (Appendix, Table A1). Frailty was not associated with a risk for superficial incisional SSI (OR 1.05, 95% CI 0.82-1.33, *P*=0.71), organ space SSI (OR 1.00, 95% CI 0.80-1.27, *P*=0.98), or reoperation (OR 1.17, 95% CI 0.95-1.44, *P*=0.13) (Appendix, Table A1).

Multivariate subset analysis of the frail population found that frail patients with an end colostomy had a 66% greater risk for total medical complications (OR 1.66, 95% CI 1.26-2.19, *P*<0.01) than frail patients with a primary anastomosis (Appendix, Table A2). Frail patients with an end colostomy were not associated with a risk of superficial incisional SSI (OR 1.51, 95% CI 0.87-2.62, *P*=0.15), organ space SSI (OR 0.73, 95% CI 0.38-1.39, *P*=0.34), reoperation (OR 0.62, 95% CI 0.35-1.09, *P*=0.10), or readmission (OR 1.03, 95% CI 0.71-1.48, *P*=0.89) compared to frail patients with a primary anastomosis (Appendix, Table A2).

## DISCUSSION

This analysis demonstrates that frail patients undergoing surgery for left-sided colon cancer had worse outcomes than nonfrail patients, with a greater risk for medical complications and readmission. Interestingly, frail patients were more likely to receive an end colostomy than nonfrail patients. In the frail population, receiving an end colostomy did not decrease the risk for organ space SSI, reoperation, or readmission compared to frail patients with a primary anastomosis. This finding suggests that frailty alone should not prompt the decision to perform an end colostomy. A primary anastomosis may be considered in the frail patient who does not have any other significant risk factors for anastomotic leak. Our analysis additionally showed that increasing frailty is associated with increasing rates of postoperative complications, longer hospital stays, and a greater percentage of patients requiring skilled nursing facilities at discharge, in concordance with other published studies.^14–17^

An estimated 10% to 14% of colorectal cancer surgeries result in a permanent ostomy.^21^ Patients with an ostomy report worse quality of life and social well-being.^21^ Ostomy-related problems include depression, dissatisfaction with appearance and sexual activity, gas, constipation, difficulty with clothing and travel, fatigue, and worry about ostomy noises. Ostomy-related postoperative complications, including peristomal dermatitis, parastomal hernia, stenosis, and prolapse, occur in 21% to 70% of cases.^22^

A commonly cited reason for performing an end colostomy vs a primary anastomosis is the belief that frail patients do not have the physiologic reserve to withstand the morbidity associated with an anastomotic leak. Older age, higher ASA physical status classification scores, and early (0 to 3 days) anastomotic leaks have been found to be reliable predictors of failure to rescue anastomotic leak after colectomy for colon cancer.^23,24^ The mFI-5 is a poor predictor of failure to rescue,^23^ but frail patients have been found to be more vulnerable to failure to rescue once a leak occurs.^25^ While these concerns are important, the data presented here suggest that frail patients do not have a higher rate of reoperation when controlling for other factors and may not need to receive end colostomies more frequently than nonfrail patients simply on the basis of frailty. Indeed, an end colostomy may subject frail patients to other complications, such as hernia and prolapse that would also be poorly tolerated in the setting of their decreased functional reserve. It is unlikely that all end colostomies can be avoided, as common emergency surgery indications to perform an end colostomy include perforated diverticulitis with fecal peritonitis, sepsis requiring pressor support, and large bowel obstruction. Our multivariate analysis showed that the following patient factors should also be taken into consideration when deciding whether to perform an end colostomy: smoking status, dialysis dependence, and preoperative serum albumin levels.

A 2022 ACS NSQIP study showed an increased risk of anastomotic leak in frail patients undergoing elective colectomy for colon cancer.^25^ Methodologic differences between our investigations likely account for this difference in findings. Specifically, we focused our investigation on left-sided colon cancers to focus on colocolonic and colorectal anastomoses, while the other study included all sites of colon cancer. A single-center Japanese study published in 2020 concurred with our findings and showed no association between frailty and anastomotic leak.^26^ While we were not able to specifically examine anastomotic leak with this dataset, our study used the related surrogate markers of organ space SSI and reoperation to help answer the question of whether frail patients undergoing colon surgery would benefit from an end colostomy vs primary anastomosis. We believe this study can help inform surgical decision-making for frail patients undergoing colectomy for left-sided colon cancer. However, as with other retrospective studies, additional study in the form of prospective randomized trials can help confirm the role that frailty may play in the surgical decision of end colostomy vs primary anastomosis in left-sided colon cancer.

Our study has limitations. First, we used surrogate measures to quantify anastomotic leak rates. Second, the association between procedure choice and cancer stage could not be elicited, as cancer stage is not a variable in the ACS NSQIP data user file. The frail and nonfrail groups may have had differences in cancer staging, and staging differences may have been present in the frail patient population that received an end colostomy vs primary anastomosis. Additionally, patients who receive an end colostomy usually do so for circumstances that increase the risk for anastomotic leak (ie, fecal peritonitis, perioperative sepsis, large bowel obstruction); therefore, the increased risk for medical complications and readmission among frail patients with an end colostomy may be related to other factors not accounted for in this study and not to the end colostomy itself. Finally, any database study is limited by coding and recording errors that may be present, as well as the retrospective nature of the study.

## CONCLUSION

Frail patients with left-sided colon cancer are more likely to receive an end colostomy, yet an end colostomy was not associated with a lower risk of reoperation or organ space SSI. Frailty alone should not prompt the decision to perform an end colostomy in left-sided colon cancer. Further studies are needed to guide surgical decision-making in this understudied population.

## Notes

*This article meets the Accreditation Council for Graduate Medical Education and the American Board of Medical Specialties Maintenance of Certification competencies for Patient Care, Medical Knowledge, and Practice-Based Learning and Improvement.*

## Appendix. Supplemental Tables for Impact of Frailty Upon Surgical Decision-Making for Left-Sided Colon Cancer

**Table A1.** Multivariate Analysis Identifying Factors Associated With Total Medical Complications, Superficial Incisional Surgical Site Infection (SSI), Organ Space SSI, Reoperation, and Readmission

Outcome	Variable	Odds Ratio	95% CI	*P* Value
**Total medical complications**	Age ≥65 years	1.49	1.34-1.66	<0.01
	Body mass index ≥30 kg/m^2^	0.93	0.83-1.03	0.17
	Dyspnea	1.50	1.27-1.78	<0.01
	Renal failure	2.95	1.32-6.58	<0.01
	Disseminated cancer	1.42	1.24-1.62	<0.01
	Steroid use	1.39	1.05-1.83	0.02
	Bleeding disorder	1.77	1.44-2.19	<0.01
	Preoperative serum albumin <3.5 mg/dL	2.37	2.13-2.64	<0.01
	Transfusion^a^	1.84	1.46-2.31	<0.01
	Emergency surgery	1.87	1.56-2.25	<0.01
	End colostomy	1.90	1.65-2.19	<0.01
	Frailty	1.45	1.29-1.63	<0.01
**Superficial incisional SSI**	Body mass index ≥30 kg/m^2^	1.83	1.49-2.25	<0.01
	Current smoker within 1 year	1.35	1.04-1.76	0.03
	Renal failure	3.33	1.13-9.81	0.03
	Preoperative serum albumin <3.5 mg/dL	1.70	1.36-2.12	<0.01
	End colostomy	1.87	1.46-2.47	<0.01
	Frailty	1.05	0.82-1.33	0.71
**Organ space SSI**	Current smoker within 1 year	1.39	1.09-1.77	<0.01
	Steroid use	1.39	0.84-2.31	0.20
	Bleeding disorder	1.92	1.35-2.75	<0.01
	Preoperative serum albumin <3.5 mg/dL	1.57	1.27-1.94	<0.01
	Sepsis within 48 hours prior to surgery	2.18	1.58-3.00	<0.01
	End colostomy	1.01	0.76-1.36	0.93
	Frailty	1.00	0.80-1.27	0.98
**Reoperation**	Current smoker within 1 year	1.25	1.00-1.56	0.05
	Dialysis dependent	2.38	1.25-4.52	<0.01
	Steroid use	1.42	0.91-2.22	0.12
	Preoperative serum albumin <3.5 mg/dL	1.32	1.09-1.59	<0.01
	Sepsis within 48 hours prior to surgery	1.78	1.30-2.43	<0.01
	Emergency surgery	1.59	1.16-2.18	<0.01
	End colostomy	0.90	0.68-1.19	0.45
	Frailty	1.17	0.95-1.44	0.13
**Readmission**	Age ≥65 years	1.19	1.04-1.36	0.01
	Disseminated cancer	1.44	1.21-1.72	<0.01
	Preoperative serum albumin <3.5 mg/dL	1.41	1.22-1.64	<0.01
	Sepsis within 48 hours prior to surgery	1.30	1.00-1.68	0.05
	Transfusion^a^	1.34	0.98-1.83	0.07
	End colostomy	1.10	0.90-1.35	0.34
	Frailty	1.53	1.32-1.77	<0.01

^a^Transfusion of ≥1 unit of whole/packed red blood cells within 72 hours prior to surgery.

**Table A2.** Multivariate Analysis Identifying Factors Associated With Total Medical Complications, Superficial Incisional Surgical Site Infection (SSI), Organ Space SSI, Reoperation, and Readmission in the Frail Population

Outcome	Variable	Odds Ratio	95% CI	*P* Value
**Total medical complications**	Age ≥65 years	1.62	1.29-2.02	<0.01
	Dialysis dependent	2.06	1.12-3.81	0.02
	Bleeding disorder	1.93	1.37-2.73	<0.01
	Preoperative serum albumin <3.5 mg/dL	2.46	2.02-3.00	<0.01
	Sepsis within 48 hours prior to surgery	2.31	1.57-3.39	<0.01
	Emergency surgery	1.55	1.03-2.35	0.04
	End colostomy	1.66	1.26-2.19	<0.01
**Superficial incisional SSI**	Body mass index ≥30 kg/m^2^	1.66	1.09-2.52	0.02
	Current smoker within 1 year	1.69	1.00-2.83	0.05
	End colostomy	1.51	0.87-2.62	0.15
**Organ space SSI**	Current smoker within 1 year	1.44	0.85-2.44	0.17
	Disseminated cancer	1.50	0.85-2.65	0.16
	Bleeding disorder	1.93	1.02-3.63	0.04
	Preoperative serum albumin <3.5 mg/dL	1.78	1.17-2.70	<0.01
	End colostomy	0.73	0.38-1.39	0.34
**Reoperation**	Dialysis dependent	3.46	1.56-7.64	<0.01
	Weight loss	0.54	0.23-1.27	0.16
	Preoperative serum albumin <3.5 mg/dL	1.49	1.04-2.13	0.03
	Sepsis within 48 hours prior to surgery	3.23	1.85-5.64	<0.01
	Emergency surgery	1.94	1.02-3.67	0.04
	End colostomy	0.62	0.35-1.09	0.10
**Readmission**	Age ≥65 years	1.21	0.92-1.59	0.17
	Disseminated cancer	1.50	1.05-2.13	0.03
	Preoperative serum albumin <3.5 mg/dL	1.48	1.14-1.91	<0.01
	End colostomy	1.03	0.71-1.48	0.89
